# Comparative Evaluation of U.S. Brand and Generic Intravenous Sodium Ferric Gluconate Complex in Sucrose Injection: Physicochemical Characterization

**DOI:** 10.3390/nano8010025

**Published:** 2018-01-05

**Authors:** Dajun Sun, Rodney Rouse, Vikram Patel, Yong Wu, Jiwen Zheng, Alokita Karmakar, Anil K. Patri, Priyanka Chitranshi, David Keire, Jia Ma, Wenlei Jiang

**Affiliations:** 1 Office of Research and Standards, Office of Generic Drugs, Center for Drug Evaluation and Research, U.S. Food and Drug Administration, Silver Spring, MD 20993, USA; dajun.sun@fda.hhs.gov; 2 Division of Applied Regulatory Science, Office of Clinical Pharmacology, Office of Translational Sciences, Center for Drug Evaluation and Research, U.S. Food and Drug Administration, Silver Spring, MD 20993, USA; rodney.rouse@fda.hhs.gov (R.R.); vikram.patel@fda.hhs.gov (V.P.); 3 Office of Science and Engineering Laboratories, Center for Devices and Radiological Health, U.S. Food and Drug Administration, Silver Spring, MD 20993, USA; yong.wu@fda.hhs.gov (Y.W.); jiwen.zheng@fda.hhs.gov (J.Z.); 4 National Center for Toxicological Research, U.S. Food and Drug Administration, Jefferson, AR 72079, USA; Alokita.Karmakar@fda.hhs.gov (A.K.); anil.patri@fda.hhs.gov (A.K.P.); 5 Division of Pharmaceutical Analysis, Office of Testing and Research, Office of Pharmaceutical Quality, Center for Drug Evaluation and Research, U.S. Food and Drug Administration, Saint Louis, MO 63110, USA; Priyanka.chitranshi@fda.hhs.gov (P.C.); david.keire@fda.hhs.gov (D.K.); 6 Bindley Bioscience Center, Purdue University, West Lafayette, IN 47907, USA; ma420@purdue.edu

**Keywords:** sodium ferric gluconate complex, parenteral iron, Ferrlecit, generic drugs, bioequivalence

## Abstract

The objective of this study was to evaluate physicochemical equivalence between brand (i.e., Ferrlecit) and generic sodium ferric gluconate (SFG) in sucrose injection by conducting a series of comparative in vitro characterizations using advanced analytical techniques. The elemental iron and carbon content, thermal properties, viscosity, particle size, zeta potential, sedimentation coefficient, and molecular weight were determined. There was no noticeable difference between brand and generic SFG in sucrose injection for the above physical parameters evaluated, except for the sedimentation coefficient determined by sedimentation velocity analytical ultracentrifugation (SV-AUC) and molecular weight by asymmetric field flow fractionation-multi-angle light scattering (AFFF-MALS). In addition, brand and generic SFG complex products showed comparable molecular weight distributions when determined by gel permeation chromatography (GPC). The observed minor differences between brand and generic SFG, such as sedimentation coefficient, do not impact their biological activities in separate studies of in vitro cellular uptake and rat biodistribution. Coupled with the ongoing clinical study comparing the labile iron level in healthy volunteers, the FDA-funded post-market studies intended to illustrate comprehensive surveillance efforts ensuring safety and efficacy profiles of generic SFG complex in sucrose injection, and also to shed new light on the approval standards on generic parenteral iron colloidal products.

## 1. Introduction

Among iron’s many critical roles in metabolic processes, the most essential function is in the synthesis of hemoglobin to meet the need for oxygen transport. Iron replacement medications formulated as parenteral nano-sized colloids of carbohydrate-coated ferric oxyhydroxide are an effective treatment for iron deficiency anemia in chronic kidney disease patients receiving hemodialysis and responding to erythropoiesis stimulating agents. As of May 2017, innovator iron-carbohydrate complex drug products on the U.S. market include INFed (iron dextran), Dexferrum (iron dextran), Ferrlecit (sodium ferric gluconate (SFG) complex), Venofer (iron sucrose), Feraheme (ferumoxytol), and Ferinject (ferric carboxymaltose). These iron colloidal nanoparticle products differ in the composition of their carbohydrate shells, iron core sizes and hydrodynamic radii. Nevertheless, in humans these drugs are distributed and cleared from the bloodstream through a similar process that involves phagocytosis in the reticuloendothelial system (RES) [1]. In addition, these drug products may have different clearance rates as a result of their varied compositions. The mechanism of action of these iron colloidal drugs involves uptake by cellular lysosomes in which iron ions are released from the colloidal nanoparticles and become part of the intracellular labile iron pool for use in biological processes [2].

The availability of generic drugs lowers costs and enhances access to safe and efficacious drugs for patients who need them. In this case, the presence of generic SFG may provide a more affordable treatment option for iron deficiency anemia patients intolerant to iron dextran [3] who face more costly alternatives, such as the innovator iron sucrose and SFG products. In March 2011, U.S. Food and Drug Administration (FDA) approved the first generic copy of Ferrlecit (SFG complex) among all parenteral iron-carbohydrate colloidal products. As of May 2017, the generic copy of intravenous SFG complex in sucrose injection remains the only approved generic product of intravenous iron colloidal nanoparticles in the U.S.

The generic version of SFG complex in sucrose injection was approved based on qualitative (Q1) and quantitative (Q2) formulation sameness, in vivo bioequivalence studies, and in vitro characterizations [4]. The FDA recommends generic SFG products to be Q1/Q2 the same as Ferrlecit. For in vivo bioequivalence studies, the FDA recommends a single-dose, randomized, parallel pharmacokinetic (PK) bioequivalence study in healthy subjects, with measurement of plasma total iron (TI) and transferrin-bound iron (TBI). To demonstrate bioequivalence between the brand and generic SFG complex formulations, the 90% confidence intervals of the generic drug for the maximum value of the difference in concentration and area-under-the-curve between TI and TBI over all time points measured should be within an 80.00% to 125.00% range of innovator product’s values [5]. 

In addition to in vivo PK evidence, the FDA recommends comparative in vitro studies to illustrate formulation sameness and to predict any potential differences that may give rise to potential differences in in vivo stability and distribution patterns of the iron-carbohydrate complex. A series of comparative physicochemical characterization tests are recommended to ensure similar iron core size, iron oxide crystalline structure, iron environment, composition of carbohydrate shell, overall particle morphology, as well as comparable labile iron under physiologically relevant conditions [6]. It is recommended that these characterization tests be conducted with at least three lots of each of brand and generic products. The structural features and release profiles of iron colloids are important to ascertain compatible tissue distribution and comparable in vivo leakage of labile iron between the brand and generic SFG products. In recent years, advanced analytical techniques have become available for characterizing physicochemical properties of iron colloidal nanoparticle products such as ferumoxytol [7], iron isomaltoside [8], and iron sucrose [9] as well as the stability of SFG complex [10,11].

For example, molecular weight distribution of SFG polymers is commonly determined by size exclusion chromatography (e.g., gel permeation chromatography (GPC) [10]) and also explored with asymmetric field-flow fractionation (AFFF) [12]. The size distribution and zeta potential of SFG nanoparticles can be measured by a dynamic light scattering (DLS) particle sizing and zeta potential analyzer [10], and the iron core size can be measured by atomic force microscopy (AFM) [13] and transmission electron microscopy (TEM) [14]. More advanced analytical tools such as sedimentation velocity analytical ultracentrifugation (SV-AUC) [15] have been applied to determine the hydrodynamic radius distribution of nanoparticles.

The objective of the current study was to conduct physicochemical characterizations between Ferrlecit and its generic counterpart using state-of-the-art analytical technologies in support of follow-up in vitro cellular uptake [16] and rat biodistribution [17] studies. In this report, we present inductively coupled plasma-mass spectrometry (ICP-MS), total organic carbon (TOC) measurement, thermogravimetric analysis (TGA), viscosity measurement, cryo-TEM, AFM, SV-AUC, GPC and AFFF methods for the physicochemical characterizations of the innovator and generic SFG complex products. Coupled with the ongoing clinical study comparing the labile iron level in healthy volunteers [18], the FDA-funded post-market studies intended to illustrate comprehensive surveillance efforts ensuring safety and efficacy profiles of generic SFG complex in sucrose injection, and also to shed new light on the approval standards on generic parenteral iron colloidal products.

## 2. Materials and Methods

### 2.1. Materials

Three lots of Ferrlecit and of a generic copy of sodium ferric gluconate (SFG) complex in sucrose injection products containing 62.5 mg elemental iron per 5-mL ampoule as per the drug labels were purchased from Sanofi U.S. LLC (Bridgewater, NJ, USA) and Watson Pharma, Inc. (Corona, CA, USA), respectively (see Table 1 for the lot number and expiration date). Filtered and deionized 18 MΩ water was supplied in house by a Millipore Milli-Q System (Bedford, MA, USA). For the gel permeation chromatography (GPC) experiments, Pullulan polysaccharide standards (6.2 kDa to 805 kDa) were purchased from Sigma-Aldrich (St. Louis, MO, USA). All other chemicals and solvents used in this study were HPLC (High Performance Liquid Chromatography) or reagent grade obtained commercially and used as received.

### 2.2. Physicochemical Properties of SFG Formulations

#### 2.2.1. Measurement of Elemental Iron Concentration by Inductively Coupled Plasma-Mass Spectrometry (ICP-MS)

The ICP-MS (NexlON 300D, Perkin Elmer, Waltham, MA, USA) measurement was conducted in the collision cell technology (CCT) mode to determine the total concentration of elemental iron in Ferrlecit and generic SFG formulations. The ICP-MS was tuned using 1 ppb Tune A solution to meet the required performance. Iron standard solution diluted with 2% HNO_3_ to 100–1000 ppb (i.e., 0.01–0.1 mg elemental iron per mL) was used as calibration standards, and an internal standard solution containing 100 ppb scandium was introduced along with samples. The background equivalent concentration (BEC) was measured to be 0.12 ppb with a detection limit of 0.009 ppb. To prepare for ICP-MS samples, 20 µL of SFG formulations were microwave digested in 3 mL of concentrated HNO_3_. Elemental iron in formulations was then quantified in triplicate using ICP-MS by spraying the samples via a glass concentric nebulizer into a cyclonic chamber at a rate of 250 μL/min in a carrier gas (H_2_) at a flow rate of 0.3 mL/min.

#### 2.2.2. Total Organic Carbon (TOC) Measurement 

Based on the recommendation in United States Pharmacopeia/National Formulary (USP/NF) [19], Ferrlecit and generic SFG samples were transferred to the reaction vessel treated with sodium persulfate, exposed to high intensity UV light and heated to 80 °C. Under the conditions, organic carbon is converted to CO_2_ which can be swept from the reaction vessel by a stream of N_2_ into a nondispersive infrared (NDIR) detector of a Shimadzu TOC-V_CSH_ Total Organic Analyzer system. The concentration of CO_2_ is correlated to the total carbon content of the sample which is measured from the standard calibration curve.

#### 2.2.3. Thermal Decomposition/Degradation by Thermogravimetric Analysis (TGA)

TGA analysis was performed on PerkinElmer TGA 4000 instrument (PerkinElmer, Waltham, MA, USA). 10 μL of Ferrlecit and generic SFG samples was heated from 30 °C to 800 °C under N_2_ at a rate of 10 °C/min, and the samples were held at 800 °C for 2 min.

#### 2.2.4. Viscometry Measurement

The viscosity of Ferrlecit and generic SFG samples was measured by a Brookfield DV-II+ viscometer (Ametek Brookfield, Middleborough, MA, USA) with 60 rpm at room temperature (25 °C) and a spindle number of 40.

### 2.3. Particle Size Distribution and Surface Charge of Nanoparticles

#### 2.3.1. Dynamic Light Scattering (DLS) and Zeta Potential

The size distribution of an iron hydroxide core surrounded by a carbohydrate shell was determined by DLS. Ferrlecit and generic SFG samples diluted 100 times with 18 MΩ purified water, 10 mM NaCl solution, and filtered saline solution (final iron concentration of 0.125 mg Fe per mL) were measured using a Zetasizer Nano ZS DLS particle sizing and zeta potential system (Malvern Instruments Ltd., Worcestershire, UK) including a laser with a wavelength of *λ* = 633 nm, which illuminated the samples and detected the scattering information at the 173° angle (Noninvasive Back-scatter technology). Zeta potential measurements of Ferrlecit and generic SFG samples diluted 50 times with 10 mM NaCl solution were performed in the same Zetasizer Nano ZS DLS particle sizing and zeta potential system (Malvern Instruments Ltd., Worcestershire, UK). The pH values were measured before and after each measurement.

#### 2.3.2. Cryogenic Transmission Electron Microscopy (cryo-TEM)

In order to investigate the size distribution and morphology of the iron hydroxide core, the cryo-TEM protocol, similar to the previously reported method [14], was performed using a Jeol 1400 TEM/STEM (scanning TEM) operated at 120 kV and viewed under the minimum dose system mode. A 2 mL aliquot of SFG complex products was first placed on a glow-discharged (EMS (electron microscopy science) 150T S) copper grid (Quantifoil R 2/1, 200 mesh) hang in a Leica EM GP grid plunge freezer (Leica Microsystem Inc., Buffalo Grove, IL, USA). The temperature and humidity of plunge freezer chamber were maintained at 25 °C and 82%, respectively. The grid was then blotted automatically for 6 sec to remove excess liquid and immediately plunged into a bath of liquid ethane at −175 °C. Images of iron cores were recorded with a digital charge-coupled device (CCD) Camera (ORIUS SC1000, Gatan, Pleasanton, CA, USA) at a nominal magnification of 100,000 and the size distribution was analyzed using Gatan Digital Micrograph software (Gatan, Pleasanton, CA, USA).

#### 2.3.3. Atomic Force Microscopy (AFM)

AFM was used to determine the size and particle morphology of Ferrlecit and generic SFG. Samples were diluted 1:20 using MilliQ water to reach a final concentration of 0.625 mg Fe per mL. 15 μL of the 1:20 solution was deposited onto a freshly cleaved mica disk (0.25 inch in diameter). After 30-s incubation, the mica surface was rinsed with water and dried by centrifugation. Each specimen was placed on the vacuum chuck of the Digital Instruments (Bruker Nano, Santa Barbara, CA, USA) NanoScope AFM system fitted with NanoScope IIIA Controller with Phase Extender Module and Dimension 3100 Large Sample AFM with type G Scanner. TappingMode™ was used to capture height and phase data types for 1 μm^2^ fields of view at a resolution of 512 × 512 pixels for images to be used for measurement. In three independent runs of specimen preparation and imaging for each lot, a total of 216 images were evaluated to analyze 2460 well-separated particles (950 for Ferrlecit lot #D2C283A, 534 for Ferrlecit lot #D2C593A, and 976 for generic SFG lot #132296.1) whose height could be readily measured.

#### 2.3.4. Sedimentation Velocity Analytical Ultracentrifugation (SV-AUC)

SV-AUC was performed to compare the size distribution based on distribution of sedimentation coefficients between Ferrlecit and generic SFG samples. Samples were diluted 50 times by mass with 1× PBS at pH 7.4 (Corning Corp., Corning, NY, USA) prior to SV-AUC analysis. An Optima XL-I SV-AUC (Beckman Coulter, Fullerton, CA, USA) with an absorbance optical detection system was used for SV-AUC. SV-AUC sample cells were assembled with a 12 mm epon double-sector centerpiece and two sapphire windows, and filled with 400 µL of sample solutions and phosphate buffer solution. The SV-AUC sample cells were placed in an An-60 Ti rotor, and the rotor was equilibrated at 20 °C in the rotor chamber for 2 h before centrifuging. The rotor was centrifuged at 20,000 rpm at 20 °C and monitored by the UV absorbance detector at 479 nm. The ls-g*(s) model from SEDFIT, which describes sedimentation profiles for non-diffusing species, was employed to analyze acquired absorbance data based on previously published method [20].

### 2.4. Molecular Weight (MW) Determination

#### 2.4.1. Gel Permeation Chromatography (GPC)

The GPC measurements were performed on a Hewlett Packard 1100 HPLC system coupled with an online refractive index detector (Optilab rEX, Wyatt Technology Corp., Santa Barbara, CA, USA). The formulations were eluted through a column which was calibrated using commercial Pullulan polysaccharide molecular weight standards (Sigma Aldrich, St. Louis, MO, USA). The standards and samples were analyzed using a refractive index detector. The samples were analyzed using two different methods described below. For both methods, the column temperature was kept at 35 °C and the injection volume was 40 µL for standards and 50 µL for samples. The auto-sampler was maintained at 20 °C during the analysis. The molecular weight of samples was calculated by means of a multivariate calibration curve generated using the Astra 6.1.1 software (Wyatt Technology Corp., Goleta, CA, USA).

Method 1: Ferrlecit and generic SFG samples were diluted 20 times with deionized water (500 μL sample diluted into a 10 mL volumetric flask) for GPC analysis. Chromatography was performed on a 300 × 8 mm Shodex OH pak^®^ SB-806M HQ column (polyhydroxy methacrylate with a particle size of 13 μm and a pore size of 1500 nm). Mobile phase consisted of 0.1 M Na_2_SO_4_ in isocratic mode at a flow rate of 0.5 mL/min. Pullulan polysaccharide standards from 6.2 kDa to 805 kDa were used for generating the calibration curve.

Method 2: Chromatography was performed on a 300 × 7.8 mm Toso Haas TSK Gel G40000SW_XL_ column (spherical silica with a particle size of 8 μm and a pore size of 25 nm). Aqueous solution of sodium azide (0.02 *w*/*v* %) and 0.01% Ferrlecit at pH 7.0 was used as the mobile phase at an isocratic flow of 0.5 mL/min. Ferrlecit and generic SFG samples were diluted 40 times with mobile phase solution. Pullulan polysaccharide standards from 21 kDa to 805 kDa were used for generating the calibration curve.

#### 2.4.2. Asymmetric Filed Flow Fractionation—Multi-Angle Laser Scattering (AFFF-MALS)

AFFF with trapezoidal channel geometry was employed with Eclipse software for instrument control (Wyatt Technology Corporation, Santa Barbara, CA, USA) with Agilent 1200 HPLC pumps and UV detector (Agilent Technologies, Inc., Santa Clara, CA, USA). 100 mM NaNO_3_ was used as the mobile phase. The accumulation wall consisted of a 10 kDa regenerated cellulose filter. An 18-angle DAWN HELEOS II detector and Optilab T-rEX differential refractometer (Wyatt Technology Corporation, Santa Barbara, CA, USA) operating at a wavelength of 660 nm was calibrated with high purity toluene (Sigma-Aldrich, St. Louis, MO, USA). The Optilab T-rEX differential refractometer used for online concentration measurements was calibrated with NaCl. Astra v6.1.4 software (Wyatt Technology Corporation, Santa Barbara, CA, USA) was used for evaluations of light scattering and refractive index data. The molar mass and size calculated are based on Debye plots using the linear Zimm method.

## 3. Results

### 3.1. Comparative In Vitro Characterization of SFG Formulations

An elemental analysis of iron and organic carbon, thermogravimetric analysis (TGA) and viscosity were conducted to assess the key formulation components of Ferrlecit (two lots D2C283A and D2C593A) and the generic SFG (one lot 132296.1). Based on the drug label [21], each vial of 5 mL of SFG complex contains 62.5 mg (12.5 mg/mL) of elemental iron as sodium ferric carbohydrate complex in an aqueous solution containing 975 mg (195 mg/mL) of sucrose and 9 mg (1.8 mg/mL) of benzyl alcohol. Iron and organic carbon of Ferrlecit and the generic SFG were measured by ICP-MS and NDIR, respectively. Both drug products had comparable total elemental iron (12.4–13.2 mg/mL, translating to 99–106% of the label claim) and organic carbon (2.9–3.2 wt %) content as shown in Table 2.

**Figure 1:**
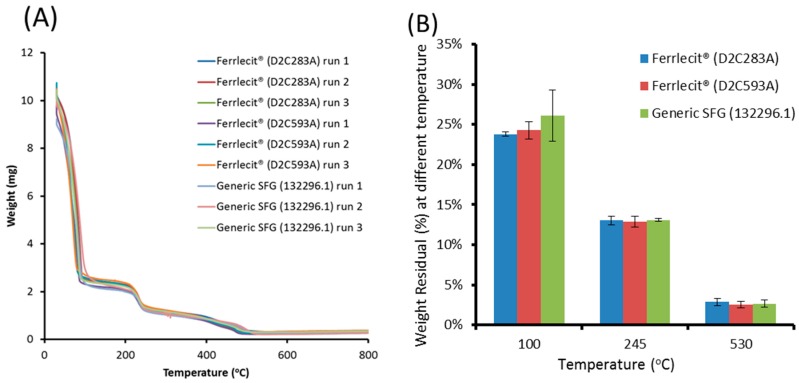
(**A**) Thermogravimetric diagrams of three independent runs of two Ferrlecit lots (D2C283A and D2C593A) and one generic SFG lot (132296.1) and (**B**) the average percentages and standard deviation (*n* = 3) of their weight residues at 100, 245 and 530 °C.

TGA was conducted to characterize the mass loss of Ferrlecit and generic SFG formulations due to oxidative decomposition. As shown in Figure 1, there were three distinct thermal events in each of the samples: from room temperature to 100 °C (~77 wt % loss), 100-245 °C (~10 wt % loss), and 245–530 °C (~10 wt % loss), leaving a weight residue of approximately 3 wt %. The ~77% weight loss from ambient temperature to 100 °C is due to water evaporation. The next ~10% weight loss at 100–245 °C fell in a similar temperature range of sucrose degradation [22]. The last ~10% weight loss at 245–530 °C seems to be the result of simultaneous oxidation and decomposition of gluconate due to its temperature coincidence with the temperature reported in the literature for free polysaccharides [23]. No significant difference in thermal events or residual mass% between Ferrlecit and the generic SFG was observed. In addition, Table 3 summarizes the viscometry results of Ferrlecit and the generic SFG measured in triplicate by using deionized water as a blank. All three samples have similar Brookfield viscosity with the value of approximately 0.88 cps at 23 °C at a spindle speed of 60 rpm.

### 3.2. Comparative In Vitro Characterization of SFG Colloidal Nanoparticles

Dynamic light scattering (DLS), cryogenic transmission electron microscopy (cryo-TEM) and atomic force microscopy (AFM) were used to determine the particle size distribution of SFG colloidal nanoparticles. As shown in Table 4, the DLS analysis shows that Ferrlecit and generic SFG have comparable z-average (10.5–12.8 nm), intensity-weighted (12.1–15.8 nm) and volume-weighted (8.1–9.5 nm) diameters of iron colloidal nanoparticles diluted 100 times with water, NaCl and saline buffer. The hydrodynamic (z-average) diameters (10.5–12.8 nm) of SFG samples measured in this study was slightly different from the reported values of 8.6 nm [8] and 10 nm [10], possibly due to different DLS methods and experimental conditions. The choice of dilution media had minimal impact on particle size measurements of intravenous SFG complex in sucrose injection but dilution between 50× to 100× is suitable for these samples. In addition, the zeta-potential analysis demonstrated that Ferrlecit and generic SFG possess negligible differences at a pH value close to the physiological pH (~pH 7.2) (Table 5). Both the zeta potential and pH levels are similar between two iron products.

**Figure 2:**
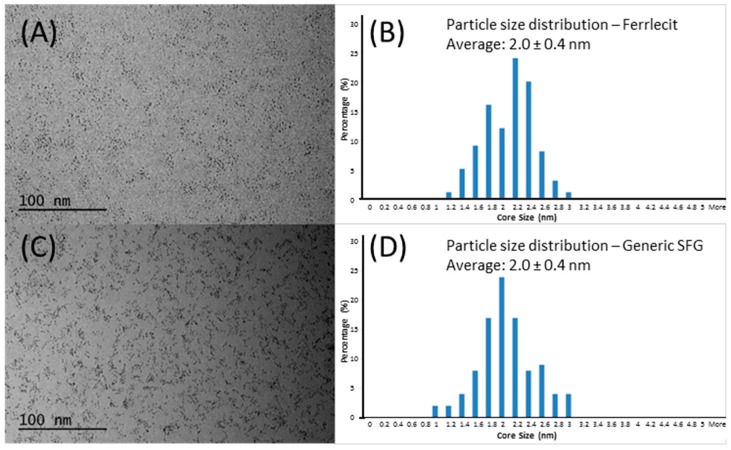
Cryogenic transmission electron microscopy (cryo-TEM) images and size distribution of Ferrlecit lots of D2C283A and D2C593A (**A**,**B**) and generic SFG lot of 132296.1 (**C**,**D**).

Both cryo-TEM and AFM analyses measured the iron core size of Ferrlecit and generic SFG. In order to investigate the size distribution and morphology of the iron hydroxide core, the cryo-TEM protocol similar to the previously reported method [14] was performed. As shown in Figure 2A,C, the dark particles in cryo-TEM images indicate the iron cores with high atomic number that absorb or scatter electrons. Both Ferrlecit and generic SFG consist of dispersed spherical nano-sized iron colloids which have an average size of approximately 2 nm with a narrow size distribution in the native state (Figure 2B,D), which is consistent with the reported value [13,14].

**Figure 3:**
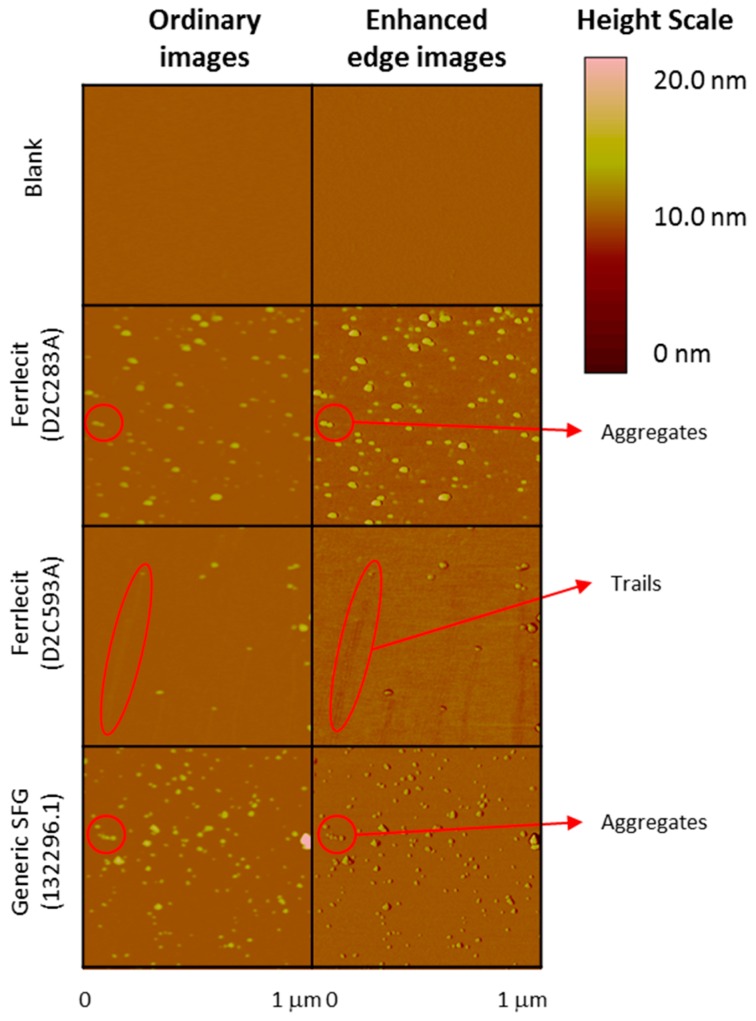
Topographic atomic force microscopy (AFM) ordinary and enhanced edge images of Ferrlecit (D2C283A and D2C593A) and generic SFG (132296.1).

**Figure 4:**
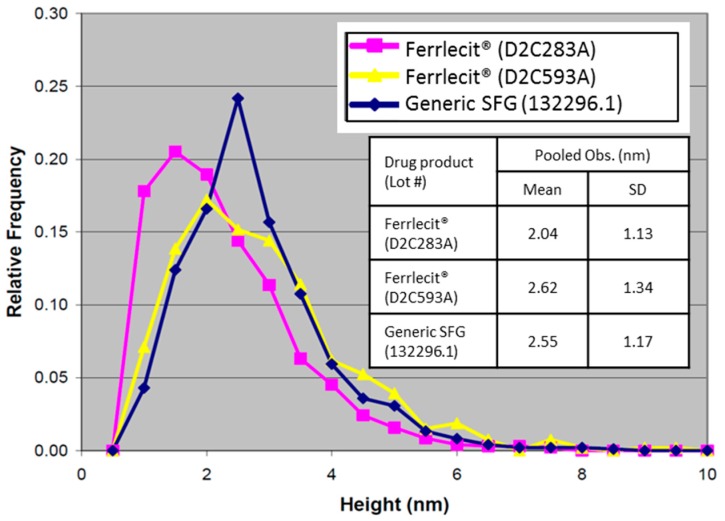
Histograms of the AFM-depth intensities as a function of particle height. Inset: pooled average of particle heights with the standard deviation for Ferrlecit D2C283A (*n* = 950), Ferrlecit D2C593A (*n* = 534), and generic SFG 132296.1 (*n* = 976).

Moreover, AFM was used to characterize particle morphology of SFG nanoparticles. Representative AFM ordinary and enhanced edge images from Ferrlecit and generic SFG lots are displayed with the same height scale (20 nm) in Figure 3. A typical water blank image confirms an example control scan for false positives. The asymmetric ellipsoidal shape of particles most likely reflects the 3-dimensional structure of the probe tip because the shape repeats for nearly every bump. Therefore, the particle height rather than width was used to estimate the particle size. Trails (see images of Ferrlecit D2C593A in Figure 3) are commonly produced when the tapping tip dislodges a weakly bound particle, slightly shifting its position on the mica by a lateral force. The inconsistent stability of particle mobility is evidence that adhesion may vary from spot to spot on the mica. The analysis of trailing particles was manually deleted if any portion of the trails was counted by the automated software. Few aggregated particles forming chains in the AFM images were also observed. Figure 4 shows the histograms of pooled average (all three runs) of AFM-depth intensities of two brand lots and one generic lot SFG as a function of particle heights. The pooled observed particle heights of Ferrlecit (D2C283A), Ferrlecit (D2C593A) and generic SFG (132296.1) were 2.04 ± 1.13, 2.62 ± 1.34, 2.55 ± 1.17 nm, respectively (Figure 3 inset), which is consistent with the reported value of 2 ± 1 nm [13]. In general, both Ferrlecit and generic SFG iron colloids predominantly consist of individual particles at similar size in a single-peaked distribution. A small fraction of the particles are larger than 5 nm, possibly due to existence of aggregated particles in both products.

**Figure 5:**
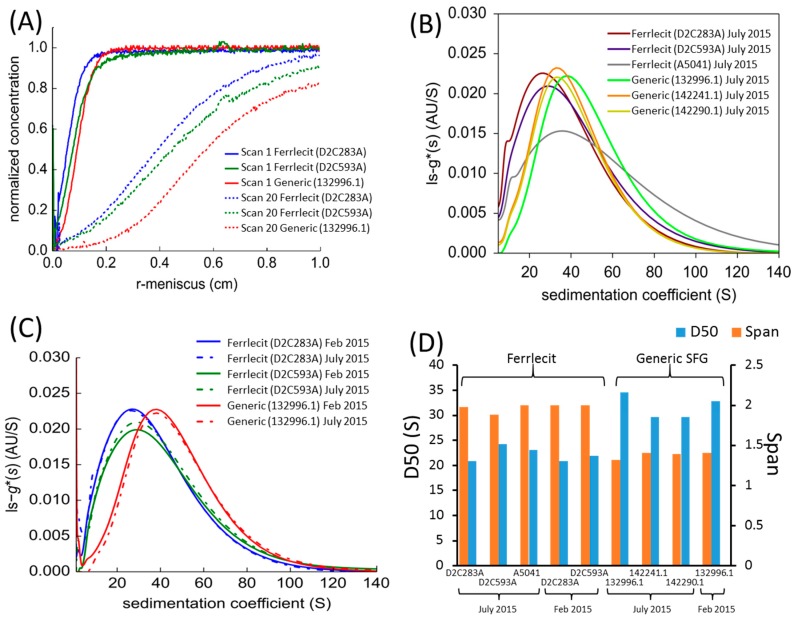
Sedimentation velocity analytical ultracentrifugation (SV-AUC) (**A**) raw data scans at 479 nm for Ferrlecit and generic SFG samples after 50-fold dilution with saline solution at 20,000 rpm. The first and twentieth scans thereafter are shown. (**B**) Normalized sedimentation coefficient distribution of Ferrlecit lots D2C283A, D2C593A and A5041 and generic SFG lots 132996.1, 142241.1 and 142290.1. (**C**) Normalized sedimentation coefficient distribution of two Ferrlecit lots D2C283A and D2C593A and one generic SFG lot 132996.1 at two time points, and (**D**) D50 and span comparison of normalized sedimentation coefficient distribution.

Sedimentation velocity analytical ultracentrifugation (SV-AUC) was used to measure the sedimentation coefficients of iron colloidal complexes of Ferrlecit and generic SFG, characterizing their macromolecular heterogeneity and particle association and aggregation. The 1st and 20th absorbance scans of three representative Ferrlecit and generic SFG samples were plotted to show the molecular concentration changes under the centrifugal forces (Figure 5A). The difference between the 1st to the 20th absorbance scans illustrates a transition from a uniformly distributed concentration across the cell to a meniscus region with depleted concentration due to the centrifugal force. The collected absorbance data were transformed into the sedimentation coefficient distributions, c(s), using the previously established method [20] and the analytical software SEDFIT (http://www.analyticalultracentrifugation.com/default.htm). Figure 5B shows the sedimentation coefficient distributions of three lots of each of Ferrlecit and generic SFG, among which two Ferrlecit lots and one generic SFG lot were tested at two time points (Figure 5C). The D50 and span of each sedimentation coefficient distribution for all the tested SFG samples were calculated to characterize the mono-modal distribution as shown in Figure 5D. In general, the iron colloidal complexes in generic SFG formulation had a higher D50 (i.e., higher sedimentation coefficient) and a lower span (i.e., a narrower peak) of the sedimentation coefficient distribution than Ferrlecit. Two Ferrlecit lots (D2C283A and D3C593A) and one generic SFG lot (132996.1) showed similar D50 and span in their sedimentation coefficient distributions over time, confirming the sample stability and method reproducibility.

### 3.3. MW Determination

As described in Section 2.4.1, the molecular weights (M_n_ and M_w_) of the sodium ferric gluconate complex in Ferrlecit and generic SFG were determined by gel permeation chromatography (GPC) using a refractive index detector applying two different methods. The different molecular weight ranges for the ferric gluconate component measured by two independent labs using two methods are reported in Table 6. Based on Method 1 described in Section 2.4.1, the GPC results show that the M_w_ of polynuclear iron oxyhydroxide in Ferrlecit and generic SFG was 25.1–36.5 kDa and 18.3–19.0 kDa, respectively. In this case, the majority of M_w_ for both Ferrlecit and generic SFG fell within the molecular weight range of 17–28 kDa, except for the Ferrlecit lot A5075 (M_w_ = 36.5 kDa). Lab 2 has identified that the percent relative standard deviation of the triplicate samples of three lots of Ferrlecit and generic SFG was 0.7–2.6% and 0.6–1.8%, respectively. Based on Method 2 described in Section 2.4.1, the GPC results show that the M_w_ of polynuclear iron oxyhydroxide in Ferrlecit and generic SFG was 384.7–467.7 kDa and 363.7–387.4 kDa, respectively. In this case, the majority of M_w_ for both Ferrlecit and generic SFG fell within the molecular weight range of 363–393 kDa, except for the Ferrlecit lot A5075 being slightly higher than the labeled range (M_w_ = 467.7 kDa). The percent relative standard deviation of the triplicate samples of three lots of Ferrlecit and generic SFG was 0.5–1.3% and 0.5–1.5%, respectively, based on Method 2. The polydispersity index (PDI) (defined as M_w_/M_n_) calculated using GPC data based on Method 1 of Ferrlecit (PDI = 2.5–3.3) is slightly larger than the generic SGF complex product (PDI = 1.9–2.2), whereas the PDI based on Method 2 (PDI = 1.1–1.2) did not exhibit noticeable differences between two products.

In addition, the number-average molecular weight (M_n_) and weight-average molecular weight (M_w_) of Ferrlecit and generic SFG were determined by asymmetrical flow field-flow fractionation (AFFF) coupled with multi-angle light scattering (MALS) as shown in Table 7. The AFFF-MALS results show that the polynuclear iron oxyhydroxide of the generic SFG had M_n_ and M_w_ of 218.4–222.2 kDa and 415.6–417.7 kDa, respectively, which were greater than those of two Ferrlecit lots (M_n_: 83.5–98.9 kDa, M_w_: 316.7–330.7 kDa). However, the PDI of generic SFG was approximately 1.9, which was smaller than that of Ferrlecit (3.3–3.8). Three independent runs of each SFG lot confirmed reproducible results.

## 4. Discussion

To gain approval of Abbreviated New Drug Applications (ANDA) in the U.S., generic iron complex products need to meet compendial or other regulatory quality standards from chemistry, manufacturing and control perspectives. Additionally, at least three lots of each of generic and brand iron complex products should be used to demonstrate comparable physicochemical characteristics of iron core, carbohydrate shell, particle size and morphology, and labile iron. Due to a limited number of commercially available Ferrlecit and generic SFG lots on the market at a given time, the current study has measured the elemental iron and carbon content, thermal properties, viscosity, particle size (DLS and AFM) and zeta potential of two Ferrlecit lots and one generic SFG lot to evaluate whether or not the brand-to-generic difference is greater than the inter-batch variability of the brand product. The sedimentation coefficient and molecular weight of three lots of each generic and brand SGF complex products were determined in order to evaluate the observed differences between brand and generic products. Previous post-marketing studies have shown comparable iron content and molecular weights [11] and labile iron levels in formulations [24] between Ferrlecit and its approved generic copy on the U.S. market. The results obtained from this study further assess physicochemical equivalence between brand and generic SFG products at the formulation, nanoparticle and polymer levels. At the formulation level, there is no noticeable difference in iron and carbon content (Table 2), viscosity (Table 3) and decomposition profiles, and residual mass based on the TGA analysis (Figure 1), indicating a comparable chemical composition.

At the nanoparticle level, the size of iron colloidal nanoparticles has an important implication for the core surface area available for dissociation and release of the reduced ferrous iron from the colloidal ferric oxyhydroxide cores [25]. The brand and generic SFG products have similar size distributions of SFG complex (DLS results in Table 4) and iron core (cryo-TEM results in Figure 2 and AFM results in Figure 3 and Figure 4), but SV-AUC analysis (Figure 5) shows that generic SFG has a higher and narrower sedimentation coefficient distribution than Ferrlecit. SV-AUC is a very sensitive analytical technique to characterize nanoparticles. Although it is not possible to translate the sedimentation coefficients into molecular weight without known buoyancy properties, the sedimentation coefficient is proportional to molecular weight and hydrodynamic radius assuming that all the SFG complexes have a similar hydrodynamic shape [26]. The generic SFG has higher and narrower sedimentation coefficient distributions (Figure 5D) with a smaller inter-batch variability (Figure 5B) but a similar intra-batch variability at two time points (Figure 5C) in comparison with Ferrlecit, which is consistent with the slightly higher iron content that was still within the acceptance limits based on elemental analysis.

At the polymer level, GPC and AFFF-MALS have been used to characterize the MW distributions of SFG colloidal macromolecules. Based on the GPC results (Table 6), M_w_ values obtained by both Labs 1 and 2 using Method 1 were quite comparable between brand and generic SFG complex products; however, the M_w_ values were not consistent with the labeled M_w_ distribution of 289,000–440,000 Da based on the GPC method [21]. In contrast, the GPC analysis using Method 2 provided M_w_ distribution results consistent with the labeled value. Notably, Method 1 used a polyhydroxy methacrylate gel column which had a 33-fold larger pore size and 1.6-fold particle size compared to the silica-based gel column used in Method 2. The differences in column material, pore size and particle size result in elution at a retention time of 17–19 min with broad peaks and 12–13 min with narrow peaks, for Methods 1 and 2 respectively. Ideally, the separation of polymers in GPC should be based on their hydrodynamic size in the absence of secondary interactions between the stationary phase and the eluent. A delayed eluting peak in Method 1 may potentially underestimate the expected M_w_ of iron colloids as the molecular weights measured by GPC can be influenced by interaction between the stationary phase and iron-sugar complexes [27], which could potentially account for the observed differences of molecular weight and PDI (Method 1) between brand and generic SGF complex products. In addition, the mobile phase in two methods was very different as described in Section 2.4.1. The GPC analysis was a relative rather than an absolute method based on the assumption that the standards and the samples were structurally similar and behaving similarly in given elution conditions. It is important to choose an appropriate column and experiment conditions to conduct GPC analysis of iron complex samples. In addition, when interpreting the GPC data, caution should be taken to understand whether the data difference originates from both individual sample differences as well as the deviations in the methods used by each lab.

The AFFF-MALS analysis (Table 7) shows that generic SFG (lot 132296.1) has greater M_n_ and M_w_ and a lower PDI than Ferrlecit (lots D2C283A and D2C593A). The molecular weight data of brand and generic SFG determined by AFFF-MALS correspond with the sedimentation coefficient trend observed with the SV-AUC technique. Currently, there are no acceptance limits established for the molecular weight range of iron colloidal complex products as determined by AFFF-MALS. Both SV-AUC and AFFF-MALS analyses were conducted by contract laboratories with limited lots of brand and generic samples. Further testing on additional lots should be conducted to better understand the observed differences.

In 2011, the European Medicines Agency (EMA) initiated a discussion on non-clinical studies for generic nanoparticle iron medicinal product applications, and the results of which were later adopted as the reflection paper on the data requirements for generic intravenous iron-based nano-colloidal products [28]. The non-binding data requirements at the European Union (EU) level encompass those requested by the FDA with additional recommendations such as non-clinical studies (e.g., characterization of RES uptake, biodistribution in animal models). Prior to the discussion, different iron sucrose similar (ISS) products, intended copies of iron sucrose (Venofer), for the treatment of iron deficiency anemia were approved in individual European countries via a decentralized procedure and in some Asian countries with different regulations. The emergence of safety and efficacy concerns with these approved ISS products [29,30,31] may be attributed to the less rigorous standards of those approval processes.

Post-market studies were performed with these ISS products. Table 8 summarizes comparative in vitro physicochemical characterization, non-clinical studies, and clinical adverse events reported in the literature between Venofer (iron sucrose) and ISSs developed by various manufacturers. Comparing the physicochemical characterizations conducted, except for the IS-Claris product, limited or no physicochemical characterization was conducted for most ISS products. IS-Claris (Claris Lifesciences Ltd., Ahmedabad, India) showed comparable physicochemical properties as the brand product [32,33]. When ISS products showed different Fe(III)/Fe(II) reduction potential from the brand product, differences in cellular uptake (e.g., iron sucrose AZAD) [34], oxidative stress and inflammatory response in rats (e.g., six ISSs in Asia) [35], hemodynamic and tissue responses in rats (e.g., Generis) [36,37], and clinical responses (e.g., Ferex) [29] were observed. In the Ferex case, there was an increase in incidences of injection site reactions and phlebitis in postpartum and gynecologic operative patients [29]. The above ISS (iron sucrose similar) products failed to meet the Iron Sucrose Injection USP (United States Pharmacopeia) standard of Fe(III)/Fe(II) reduction potential (−700 to −800 mV [38]) as an indicator of the labile iron level in the formulation, which is a critical quality attribute impacting in vivo performance of iron sucrose complex products. Switching from the originator’s product (Venofer) to ISS (Mylan SAS, Meyzieu, France) led to destabilization of a well-controlled population [30] but again no physicochemical characterizations were conducted for this generic iron sucrose complex product.

Comparing the cases of generic SFG in the U.S. and ISS in Europe and Asia, it is evident that at least some physicochemical quality parameters are sensitive predictors of in vivo outcomes for iron complex drug products. Physicochemical characterizations are critical to cross-compare the quality attributes between brand and generic parenteral iron colloidal products, as part of the bioequivalence approach ensuring equivalent efficacy, safety and stability profiles. Regulators recommend clinically relevant parameters or characteristics of nano-sized iron products to support generic drug approval [4,28]; generic drug developers, in turn, apply the most appropriate analytical techniques to establish pharmaceutical comparability. It is acknowledged that there are often multiple analytical methods that can characterize the same property of iron-carbohydrate complex products (e.g., cryo-TEM and AFM measuring the iron core size), allowing for cross-method comparison. In addition, since the method parameters can sometimes impact the resulting measurements (e.g., MW determination by GPC), product-specific methodology with an adequately discriminatory power needs to be developed and validated. The applicability of innovative methods (e.g., AFFF-MALS) should be critically assessed. Occasionally, interpreting the characterization data in the context of bioequivalence has become a regulatory challenge as the sensitivity of analytical techniques has steadily and continuously improved over time (e.g., using SV-AUC to compare Ferrlecit and generic SFG). Therefore, the results obtained from physicochemical characterization of iron-carbohydrate complex products need to be evaluated in the context of totality of evidence including in vivo bioequivalence data, formulation, manufacturing process and other information. Bearing the above considerations in mind, generic drug companies are recommended to characterize their proposed generic formulations and the brand products with a meaningful and comprehensive data set based on regulator’s guidance of key quality attributes and with multiple complementary analytical techniques and specified methodology.

In the grand scheme of generic product approval, the FDA actively conducts post-market surveillance studies in addition to pre-market evaluation. Any clinical concerns such as adverse events associated with generic SFG have been closely monitored via FDA Adverse Event Reporting System (FAERS) and the sponsor’s post-market commitments. To date, there has been no evidence suggesting any substitutability issues between brand and generic SFG in the U.S. In addition, FDA-initiated research will evaluate brand-to-generic equivalence, especially the labile iron level, of parenteral SFG complex products in human subjects. The follow-up cellular uptake [16] and biodistribution [17] studies were conducted to confirm that the differences observed in some of the physicochemical tests between Ferrlecit and generic SFG do not impact the in vivo biological activities.

## 5. Conclusions

Given growing skepticism toward iron sucrose and similar products in Europe, albeit via decentralized procedure and less rigorous approval standards, the FDA’s Office of Generic Drugs has been conducting a series of post-market investigations to proactively monitor brand-to-generic equivalence of parenteral SFG complex products, being the first approved generic version of a parenteral iron-carbohydrate colloidal product in the U.S. As part of comprehensive surveillance efforts, the current study compared elemental iron and carbon content, thermal property, viscosity, particle size, zeta potential, sedimentation coefficient and molecular weight between Ferrlecit and generic SFG. The physicochemical tests show comparable quality attributes between two therapeutically equivalent SFG products at formulation, nanoparticle and polymer levels, except for slight differences observed in SV-AUC and AFFF-MALS analyses. The evaluation of complex drug products containing iron nanomaterials using advanced analytical methods needs to be carefully considered in the context of a weight-of-evidence approach. Additional assessments, including in vitro cellular uptake in human macrophages and biodistribution studies in a rat model, confirm that the slight differences observed between Ferrlecit and generic SFG in the present study do not impact their biological activities.

## Figures and Tables

**Table 1:** Lot number and expiration date of Ferrlecit and the generic product of sodium ferric gluconate (SFG) complex in sucrose injection used in this study. Date: Month/Year.

Ferrlecit (Brand Product)		Generic Sodium Ferric Gluconate (SFG)	
Lot Number	Expiration Date	Lot Number	Expiration Date
D2C283A	10/2015	132296.1	11/2015
D2C593A	11/2015	142241.1	09/2016
A5075	09/2018	142290.1	11/2016

**Table 2:** Elemental iron and total organic carbon of two Ferrlecit lots (D2C283A and D2C593A) and one generic SFG lot (132296.1).

Drug Product (Lot #)	Elemental Fe Conc. in Formulations (mg/mL)	Total Organic Carbon (wt %)
Ferrlecit (D2C283A)	12.64 ± 0.12	3.2%
Ferrlecit (D2C593A)	12.38 ± 0.18	3.1%
Generic SFG (132296.1)	13.23 ± 0.23	2.9%

**Table 3:** Viscosity results of two Ferrlecit lots (D2C283A and D2C593A) and one generic SFG lot (132296.1) at 23 °C at a spindle speed of 60 rotations per minute (rpm).

Drug Product (Lot #)	Viscosity (cps) with 60 rpm at 23 °C
Ferrlecit (D2C283A)	0.88 ± 0.01
Ferrlecit (D2C593A)	0.87 ± 0.01
Generic SFG (132296.1)	0.88 ± 0.01

**Table 4:** Dynamic light scattering (DLS) analysis—z-average, intensity-weighted and volume-weighted diameters of iron colloidal nanoparticles in Ferrlecit and generic SFG diluted with 18 MΩ H_2_O, 10 mM NaCl, and saline buffer.

Drug Product (Lot #)	Diluent	Z-Average Diameter (nm)	Intensity-Weighted Diameter (nm)	Volume-Weighted Diameter (nm)	PDI
Ferrlecit (D2C283A)	18 MΩ H_2_O	12.7	15.8	8.4	0.208
Ferrlecit (D2C593A)	18 MΩ H_2_O	12.8	15.7	9.5	0.177
Generic SFG (132296.1)	18 MΩ H_2_O	11.3	13.3	8.2	0.173
Ferrlecit (D2C283A)	10 mM NaCl	11.9	14.1	8.7	0.148
Ferrlecit (D2C593A)	10 mM NaCl	12.5	14.1	9.2	0.156
Generic SFG (132296.1)	10 mM NaCl	11.0	12.8	8.4	0.138
Ferrlecit (D2C283A)	Saline	11.5	13.9	9.0	0.163
Ferrlecit (D2C593A)	Saline	12.1	14.5	8.8	0.158
Generic SFG (132296.1)	Saline	10.5	12.1	8.1	0.123

**Table 5:** Zeta potential and pH levels of Ferrlecit (D2C283A and D2C593A) and generic SFG (132296.1).

Drug Product (Lot #)	Zeta Potential (mV)	pH
Ferrlecit (D2C283A)	−7.95	7.23
Ferrlecit (D2C593A)	−6.77	7.25
Generic SFG (132296.1)	−7.89	7.25

**Table 6:** Gel permeation chromatography (GPC) results of number-average molecular weight (M_n_), weight-average molecular weight (M_w_), and polydispersity index (M_w_/M_n_) of three Ferrlecit lots and three generic SFG lots using two methods (Section 2.4.1) conducted by two independent labs.

Drug Product (Lot #)	Method 1 by Lab 1			Method 1 by Lab 2			Method 2 by Lab 2		
	M_n_ (kDa)	M_w_ (kDa)	PDI	M_n_ (kDa)	M_w_ (kDa)	PDI	M_n_ (kDa)	M_w_ (kDa)	PDI
Ferrlecit (D2C283A)	10.1 ± 0.5	25.1 ± 0.7	2.5	8.0 ± 0.1	22.4 ± 0.2	2.8	325.6 ± 3.9	384.7 ± 5.1	1.2
Ferrlecit (D2C593A)	10.1 ± 0.6	26.7 ± 0.8	2.6	9.1 ± 0.0	25.1 ± 0.5	2.7	332.5 ± 1.4	393.4 ± 1.9	1.2
Ferrlecit (A5075)	-	-	-	11.2 ± 0.2	36.5 ± 0.9	3.3	383.2 ± 2.1	467.7 ± 3.0	1.2
Generic SFG (132996.1)	8.9 ± 0.2	18.3 ± 0.8	2.1	9.6 ± 0.1	19.0 ± 0.1	2.0	350.6 ± 3.1	387.4 ± 2.1	1.1
Generic SFG (142241.1)	-	-	-	8.4 ± 0.6	18.3 ± 0.3	2.2	324.9 ± 2.0	365.9 ± 5.4	1.1
Generic SFG (142290.1)	-	-	-	9.6 ± 0.1	18.7 ± 0.3	1.9	327.9 ±4.1	363.7 ± 1.9	1.1

**Table 7:** Asymmetric field flow fractionation-multi-angle light scattering (AFFF-MALS) results of number-average molecular weight (M_n_), weight-average molecular weight (M_w_), and polydispersity index (PDI) of two Ferrlecit lots (D2C283A and D2C593A) and one generic SFG lot (132296.1) in three independent runs.

Drug Product (Lot #)	Run	M_n_ (kDa)	M_w_ (kDa)	PDI
Ferrlecit (D2C283A)	1	83.5 ± 2.3	316.7 ± 0.9	3.8
Ferrlecit (D2C283A)	2	88.8 ± 2.6	317.8 ± 1.3	3.6
Ferrlecit (D2C283A)	3	87.4 ± 2.1	319.1 ± 1.3	3.6
Ferrlecit (D2C593A)	1	98.9 ± 1.5	329.1 ± 0.7	3.3
Ferrlecit (D2C593A)	2	92.7 ± 2.4	329.9 ± 1.6	3.6
Ferrlecit (D2C593A)	3	92.7 ± 2.5	330.7 ± 1.3	3.6
Generic SFG (132296.1)	1	218.4 ± 0.7	415.6 ± 1.2	1.9
Generic SFG (132296.1)	2	219.6 ± 0.7	418.3 ± 1.3	1.9
Generic SFG (132296.1)	3	222.2 ± 0.7	417.7 ± 1.3	1.9

**Table 8:** Comparative in vitro physicochemical characterizations, non-clinical studies and clinical adverse events between Venofer (iron sucrose) and various Iron Sucrose Similar (ISS) products in the literature.

ISS Product (Manufacturer)	In Vitro Physicochemical Characterizations	Non-Clinical Studies (In Vitro Cellular Uptake or Biomarkers)	Clinical Adverse Events (AEs)	Ref.
IS-Claris (Claris Lifesciences Ltd., Ahmedabad, India)	Comparable results of GPC, MALDI-TOF, UV-Vis, XRD, proton and ^13^C NMR, FTIR, TGA, labile iron release, elemental analysis, Mössbauer, Raman spectroscopy, and particle size distribution	Not available (N/A)	N/A	[32,33]
Iron sucrose AZAD (AZAD Pharma, Toffen, Switzerland)	Comparable redox-active iron in formulations	Comparable iron uptake in human THP-1 and HepG2 cells	N/A	[34]
Six ISSs marketed in Asia	Comparable physical appearance	Different serum iron and transferrin saturation levels, labile iron, oxidative stress and inflammatory markers and antioxidant enzymes in the liver, heart and kidneys in rats	N/A	[35]
	Different Fe(III)/Fe(II) reduction potential, pH, titratable alkalinity, turbidity point, MW, and PDI			
Generis (Generis Co., Amadora, Portugal)	Comparable MW, pH, titratable alkalinity and physical appearance	Different systolic blood pressure, serum iron, transferrin saturation, liver enzymes, and biomarkers in liver, heart and kidney (TNF-alpha and IL6) in rats	N/A	[36,37]
	Different Fe(III)/Fe(II) reduction potential and turbidity point			
Ferex (SejongPharma, Incheon, South Korea)	Comparable pH, titratable alkalinity, turbidity point, MW, and physical appearance	N/A	There were more AEs associated with ISS than Venofer in postpartum and gynecologic operative patients.	[29]
	Different Fe(III)/Fe(II) reduction potential			
FerMed (Medice Arzneimittel Pütter GmbH, Iserlohn, Germany)	N/A	N/A	Three patients who previously tolerated with Venofer experienced urticaria, edema and headache when switching to ISS.	[31]
ISS (Mylan SAS, Saint-Priest, France)	N/A	N/A	Switching from Venofer to ISS led to destabilization of a well-controlled population of hemodialysis patients.	[30]

GPC: gel permeation chromatography; MALDI-TOF: matrix-assisted laser desorption/ionization-time-of-flight mass spectroscopy; UV-Vis: ultraviolet-visible spectroscopy; XRD: X-ray diffraction; NMR: nuclear magnetic resonance spectroscopy: FTIR: Fourier-transformed infrared spectroscopy; TGA: thermogravimetric analysis.
